# LncRNA profiles from Notch signaling: Implications for clinical management and tumor microenvironment of colorectal cancer

**DOI:** 10.3389/fimmu.2022.953405

**Published:** 2022-07-25

**Authors:** Qin Dang, Zaoqu Liu, Yang Liu, Wenkang Wang, Weitang Yuan, Zhenqiang Sun, Lin Liu, Chengzeng Wang

**Affiliations:** ^1^ Department of Colorectal Surgery, The First Affiliated Hospital of Zhengzhou University, Zhengzhou, China; ^2^ Henan Institute of Interconnected Intelligent Health Management, The First Affiliated Hospital of Zhengzhou University, Zhengzhou, China; ^3^ Department of Interventional Radiology, The First Affiliated Hospital of Zhengzhou University, Zhengzhou, China; ^4^ Department of Radiotherapy, Affiliated Cancer Hospital of Zhengzhou University, Henan Cancer Hospital, Zhengzhou, China; ^5^ Department of Breast Surgery, The First Affiliated Hospital of Zhengzhou University, Zhengzhou, China; ^6^ Department of Ultrasound, The First Affiliated Hospital of Zhengzhou University, Zhengzhou, China

**Keywords:** colorectal cancer, LncRNA, notch, prognosis, immunotherapy, tumor microenvironment

## Abstract

The interplay between long non-coding RNAs (lncRNAs) and the Notch pathway involves a variety of malignancies. However, Notch-derived lncRNAs and their latent clinical significance remain elusive in colorectal cancer (CRC). In this study, we introduced a framework that could screen Notch-derived lncRNAs (named “NLncer”) and ultimately identified 24 NLncers. To further explore the clinical significance of these NLncers, we performed LASSO and Cox regression in TCGA-CRC cohort (n = 584) and then retained six lncRNAs tightly associated with prognosis. The final model (termed “NLncS”) was subsequently tested in GSE38832 (n = 122), GSE39582 (n = 573), and an in-house clinical cohort (n = 115). Ultimately, our NLncS model could serve as an independent risk factor and afford a robust performance for assessing the prognosis of CRC patients. Additionally, patients with high NLncS risk scores were characterized by upregulation of immune pathways, strong immunogenicity, abundant CD8 + T-cell infiltration, and potentially higher response rates to CTLA4 blockers, which turned out to be suitable for immunotherapy. Aiming at globally observing the characteristics of high-risk patients, somatic mutation and methylation modification analysis provide us with evidence at the genomic and transcriptomic levels. To facilitate the clinical transformability, we mined deeply into the sensitive compounds targeting high-risk individuals and identified dasatinib as a candidate agent for patients with a high Notch risk score. In conclusion, our NLncS model is a promising biomarker for optimizing the clinical management of CRC patients.

## Introduction

As one of the primary causes in tumor-related death, colorectal cancer (CRC) ranks the third most common malignant neoplasm worldwide (1, 2). CRC is often diagnosed at an advanced stage, accompanied by high postoperative recurrence and metastasis rate, which seriously threatens the health of the public. The survival of CRC patients was subjected to a series of clinical difficulties such as unresectable surgery, chemotherapy resistance, and radiotherapy side effects (3, 4). Notably, immunotherapy has shown spectacular achievements in the oncology treatment field (5, 6). However, with the rapid development, limitations of immunotherapy emerged. As proof, immunotherapy brings the desired curative effect when applied to the suitable patient subgroups (7, 8). To address this plight, researchers need to precisely identify individuals suitable for immunotherapy.

The Notch pathway, a signal transduction system ubiquitous in cellular organisms, determines the cell fate and function (9). Studies widely validated that the Notch pathway is involved in diversified aspects of oncology, including carcinogenesis, metastasis, stemness, metabolism, apoptosis, and angiogenesis (10–15). *NOTCH3* expression is closely associated with malignant phenotypes of CRC, including higher grade, the existence of lymph nodes, and distant metastasis (10). Additionally, several Notch signaling receptors could serve as therapeutic targets for breast cancer (16). Moreover, accumulating evidence has shown that the Notch pathway influences the immune system, both innate and adaptive, *via* dendritic cells and T cells (17, 18).

For decades, the identification of abundant long non-coding RNAs (lncRNAs) >200 bp has brought out their characterization as profound components in tumor biology. LncRNA molecules were commonly present in tumors as a double-edged sword of driving tumor development or inhibiting progression (19–22). Previous studies have confirmed that lncRNA could govern key ligands in the Notch pathway to achieve tumor control, such as breast cancer, renal cell cancer, and gastric cancer (23–25). Nevertheless, the comprehensive landscape of the Notch pathway-related lncRNA in CRC remains elusive, and we hope to endue Notch pathways with a novel character and clinical application through functional lncRNA analysis.

Herein, we constructed an integrated frame capable of identifying Notch-derived lncRNA drivers (termed “NLncer”). We characterized the prognostic value of the Notch-related lncRNA signature (NLncS) for CRC patients and distinguished high-risk subgroups that are suitable for immunotherapy. For verification, two independent cohorts and a clinical in-house dataset were enrolled. Subsequently, we probed the methylation levels of distinct individuals in order to find genome-level drivers that contribute to differences in outcomes. Further, based on cell line expression profiles and drug sensitivity results (CTRP and PRISM) and Connectivity Map (CMap) analysis, we recommend the anticancer drug dasatinib as a latent treatment for a CRC high-risk subgroup.

## Materials and methods

### Public dataset collection and procession

**Figure 1:**
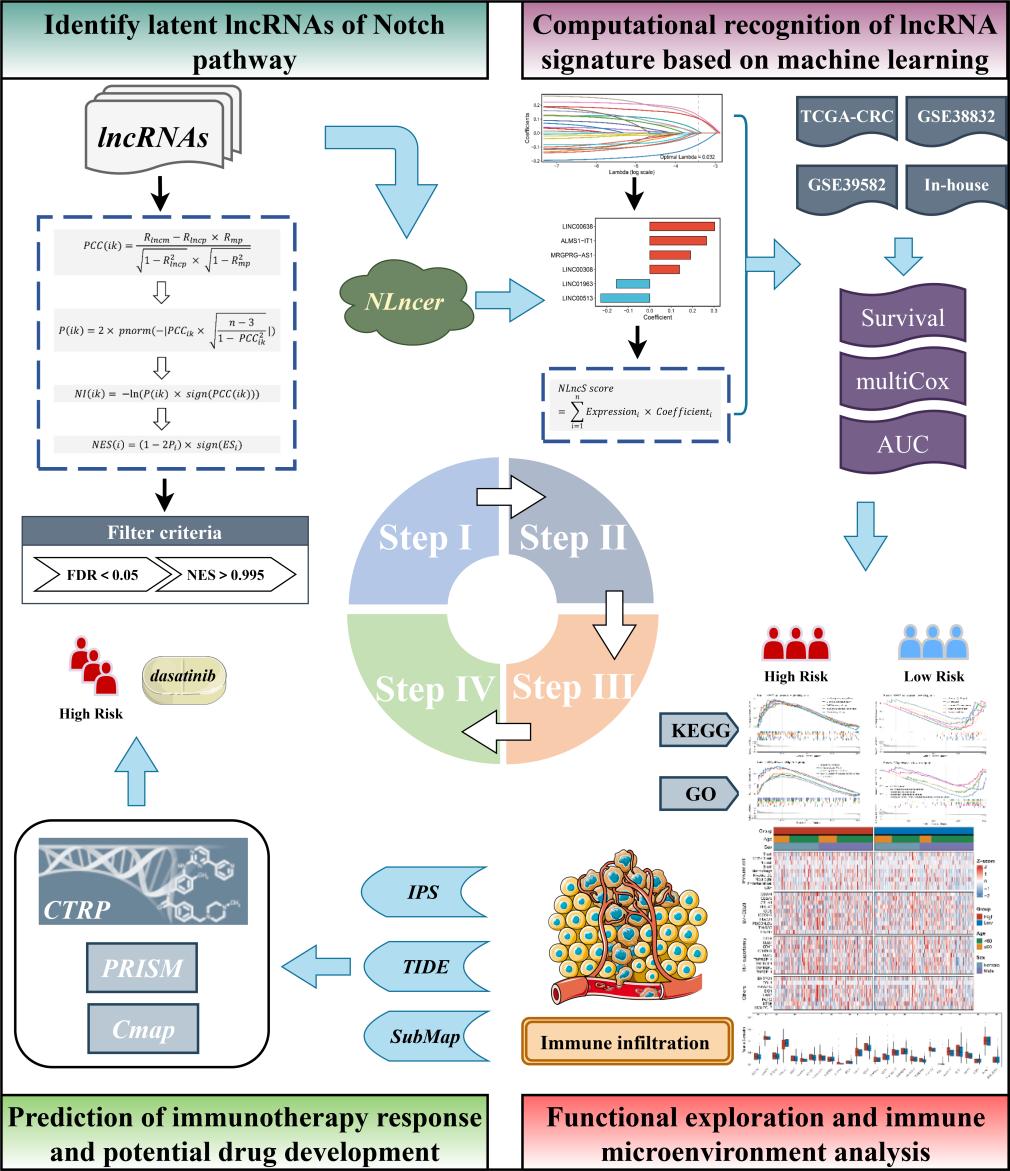
The workflow of the study.

The workflow of this research is depicted in **Figure 1**. **A** total of 1,279 CRC patients from three independent public cohorts were obtained from The Cancer Genome Atlas (TCGA, https://portal.gdc.cancer.gov) and Gene Expression Omnibus (GEO, http://www.ncbi.nlm.nih.gov/geo), including TCGA-CRC, GSE39582, and GSE38832 (**Supplementary Table 1**). We transformed the RNA-seq raw read count of TCGA-CRC to transcripts per kilobase million (TPM). The GEO datasets were collected from the Affymetrix^®^ Human Genome U133 Plus 2.0 Array (GPL570 platform) and processed by the robust multiarray averaging (RMA) algorithm with the *Affy* package. We obtained 19,526 protein-coding genes and 15,299 lncRNAs from TCGA database based on GENCODE (Homo sapiens GRCh38, https://www.gencodegenes.org/). All probes were mapped to the human genome (Hg38), and 3,439 lncRNAs were acquired by reannotating the probe sets of the GPL570 array (26). Given the batch effect, we combined the colonic adenocarcinoma (COAD) and rectum adenocarcinoma (READ) data into TCGA-CRC queues after *ComBat* algorithm-based processing. LncRNAs with an empty expression in more than half of the sample size in each cohort were excluded. Detailed baselines for three independent queues are pooled in **Supplementary Table 1**.

### NLncer: screening the potential LncRNA drivers of the Notch pathway

Aiming at discovering potential Notch-associated lncRNA drivers, we developed a comprehensive pipeline by referring to previous findings (27, 28). In brief, we arranged the mRNAs in descending order to the pertinence of particular lncRNA adjusted for tumor purity. The *fgsea* R package was used to determine whether Notch pathway-related genes were enriched. Next, the Notch enrichment score (*NES*) of all lncRNAs was measured, and those with significant *NES* were identified as Nlncers. LncRNA *i* and mRNA *k* in *n* samples were termed as *Lnc(i)* = (*lnc_1_*, *lnc_2_*,…, *lnc_n_*) and M(*k*) = (*m_1_*, *m_2_*,…, *m_n_*) in the expression matrix, respectively. The tumor purity of *n* patients was quantified *via* the *ESTIMATE* R package and termed as *p_1_*, *p_2_*,…, *p_n_*. The first-order partial correlation coefficients (PCCs) of lncRNA i and mRNA k were determined by performing the following operation:


$$PCC\left(ik\right)=\frac{R_{lncm}-R_{lncp\;}\times \;R_{mp}}{\sqrt{1-R_{lncp}^{2}}\;\times \;\sqrt{1-R_{mp}^{2}}}$$

*R_lncm_*, *R_lncp_*, and *R_mp_* were named as the Pearson correlation coefficients of lncRNA *i* and mRNA *k*, lncRNA *i* and tumor purity *p*, and mRNA *k* and tumor purity *p*, respectively. Then, *P(ik)*, the *P*-value of *PCC(ik)*, was calculated:


$$P\left(ik\right)=2\times \;pnorm\left(-\left|PCC_{ik}\times \;\sqrt{\frac{n-3}{1-\;PCC_{ik}^{2}}}\right|\right)$$

*pnorm* is defined as the normal distribution function, and *n* is the sample size. The Notch index (*NI*) was measured:


NI(ik)= −ln(P(ik) × sign(PCC(ik)))

*sign* is a method that could realize the symbolic separation of functions. All mRNAs were sequenced according to descending *NI*, and further gene set concentration analysis (GSEA) was performed. The adjusted *P*-values and concentration scores (*ES*) of lncRNA *i* were evaluated by the *fgsea* R package and combined into a *NES*:


$$NES\left(i\right)=\left(1-2P_{i}\right)\times \;sign\left(ES_{i}\right)$$

Thereby, the *NES* was calculated to range from -1 to 1. Referring to previous findings (27, 28), lncRNAs with false discovery rate (FDR) <0.05 and *NES* absolute value >0.995 were filtrated as Notch-derived lncRNAs.

### Signature generation

Given the comparability between disparate cohorts, lncRNA expression in three cohorts was converted to z-score before Notch-related lncRNA signatures (NLncS) were generated. According to the expression profile of Notch-derived lncRNAs, univariate Cox regression was calculated among TCGA-CRC, GSE39582, and GSE38832. Aware that the rigor of multiple test corrections and the small sample size may screen out some latent lncRNAs associated with survival, lncRNAs with unadjusted *P* < 0.15 and the same hazard ratio (HR) direction were adopted to construct the NLncS in combination with LASSO regression (29). The optimal lambda was obtained when the partial likelihood deviation achieved the minimum by adopting the 10-fold cross-validation algorithm. The lncRNAs with non-zero coefficients were involved in fitting signatures. The NLncS equation was computed with the following LASSO model weighting coefficient:


$$NLncS\;score=\sum_{i=1}^{n}Expression_{i}\;\times \;Coefficient_{i}$$

*n* refers to the total of significant lncRNAs, *Expression_i_* stands for the lncRNA *i* expression, and *Coefficient_i_* replaced the matching regression coefficient.

### Clinical specimens and information collection

The ethics committee of the First Affiliated Hospital of Zhengzhou University provided consent to the study. One hundred fifteen pairs of CRC primary and normal tissues surgically resected in the First Affiliated Hospital of Zhengzhou University were included. The patient selection criteria were as follows: 1) no preoperative treatment, such as radiotherapy, chemotherapy, or targeted therapy was received; 2) no complication of any other tumors; 3) no autoimmune disease. **Supplementary Table 1** provides the record of the baseline data. Fresh specimens were obtained and frozen in liquid nitrogen at -80°C for preservation. The clinical stage was in accordance with NCCN (2019) guidelines. Each individual signed informed consent. The relevant ethical review number is 2019-KY-423.

### Quantitative real-time PCR

Tissue RNA was extracted by RNAiso Plus (Takara, China) reagent, and the quality was evaluated by NanoDrop One C (Waltham, USA). Complementary DNA (cDNA) was obtained by following the protocol of the Reverse Transcription Kit (Takara Bio, Japan). Then, quantitative real-time polymerase chain reaction (qRT-PCR) was performed using SYBR Assay I Low ROX (Eurogentec, USA) and SYBR^®^ Green PCR Master Mix (Yeason, China). Each test was repeated three times. The expression level was quantized by 2^-ΔΔCt^ mode. GAPDH serves as an internal reference for normalization. The reader is referred to **Supplementary Table 2** for primer sequence information.

### Functional enrichment

For distinguishing the differences of distinct NLncS scores, the GSEA tool and *clusterProfiler* package were used to analyze the biological pathways of Kyoto Encyclopedia of Genes and Genomes (KEGG) and Gene Ontology (GO) (30). To get a standardized concentration, we chose the permutation to 1,000. Gene sets with adjusted *P*-value <0.05 were selected as significant.

### Immune infiltration assessment

We adopted the *MCPcounter* package to explore immune cell type, stromal cell, and immune checkpoint (ICPs, recruited *B7-CD28* family, *TNF superfamily*, and others, **Supplementary Table 5**) abundance in CRC tissues (31). The correlation between these immune components and the NLncS model was further compared.

### The mutation landscape and copy number variation of CRC

Somatic mutation and copy number variation (CNV) data were downloaded from TCGA-CRC portal and cBioportal website, respectively. The *TCGAbiolinks* R package was performed to get the raw mutation file. Mutations in different patient subpopulations were analyzed and visualized based on the *maftools* and *ComplexHeatmap* R package. CNV waterfall maps of the first 10 amplification (AMP) and homozygous deletion (Homdel) chromosome fragments were visualized by the *ComplexHeatmap* package.

### Estimation of methylation drivers

The raw methylation data were obtained from HumanMethylation450 array TCGA-CRC. The global methylation level (GML) of each TCGA-CRC sample was quantified based on the average beta value of a particular probe (32). The *MethylMix* package was adopted for integration of methylation and mRNA expression data. Methylation drivers, genes that were considerably inversely correlated with expression, were used to investigate the association with NLncS.

### Evaluation of response to immunotherapy

Combining the immunophenoscore (IPS), tumor immune dysfunction and exclusion (TIDE, http://tide.dfci.harvard.edu/), and subclass mapping (SubMap) algorithms predicts responsiveness to immunotherapy (33–35). TIDE is an algorithm that integrates T-cell features to characterize immune evasion situations. The IPS Z-score was computed by assessing the scores of four immunophenotypes (antigenic presentation, effector cell, inhibitory cell, and ICP) (36). The higher IPS foreboded stronger immunogenicity of individuals. SubMap was adopted to judge the degree of similarity. The Bonferroni-corrected *P* was applied to indicate similarity, and the magnitude of the *P*-value is negatively correlated with similarity.

### Prediction of therapeutic agents

The Cancer Therapeutics Response Portal datasets (CTRP) and PRISM databases store cancer cell line (CCL) sensitivity data with over 481 and 1448 compounds, respectively. In addition, both data provide the area under the curve (AUC) of dose–response as a measure of drug sensitivity. A lower AUC predicts stronger drug responsiveness. After filtering out compounds with ≥20% missing AUC values, the K-nearest neighbor (k-NN) imputation method was employed to interpolate missing variables for the remaining compounds.

### Alternative compounds that target with high-risk groups

CMap (https://portals.broadinstitute.org/cmap/), a public online tool developed from the Broad Institute, was used to determine which drugs may have an effect on high-risk samples (37). Differential expression genes (DEGs) were obtained *via* the *limma* package. Subsequently, the top 150 upregulated and 150 downregulated genes were selected for CMap analysis. Each ranked list in CMap datasets was compared with DEGs to specify where the DEGs appeared, thus yielding a score of -100 to 100. Then the enrichment score was re-ranked; the top is strongly and positively correlated with the high risk, and the bottom is the opposite.

### Statistical analysis

All work was performed in R software (4.1.0). Pearson’s chi-square test was used for categorical variables. Continuous variables were compared adapting the Wilcoxon rank-sum test or Student’s *t* test. Spearman analysis was used to analyze the correlation between groups. The *glmnet* package was adopted to fit LASSO regression. Cox regression and Kaplan–Meier (K-M) analysis were computed based on the *survival* package. The optimal cutoff value was chosen by the *survminer* package. Survival ROC and AUC were quantified *via* the *timeROC* package. *P* < 0.05 of two tails was judged statistically significant.

## Results

### The Notch pathway was significantly enriched and correlated with clinical features of CRC

**Figure 2:**
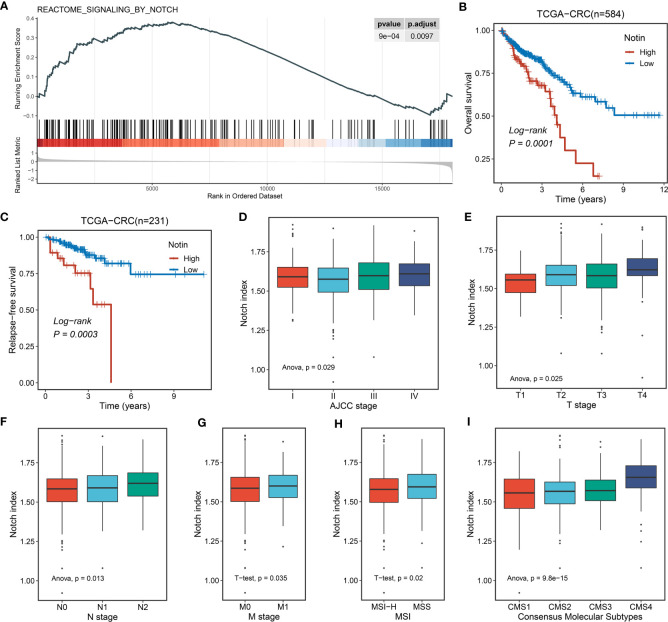
The potential biological significance of the Notch pathway in CRC. **(A)** Single-sample GSEA (ssGSEA) enrichment analysis of the Notch pathway in TCGA-CRC. **(B, C)** Kaplan–Meier curves of OS **(B)** and RFS **(C)** according to the Notch index (Notin) in TCGA-CRC. **(D-H)** Correlation between Notin and clinical features, such as AJCC stage **(D)**, T stage **(E)**, N stage **(F)**, M stage **(G)**, and microsatellite instability (MSI) state **(H)** of TCGA-CRC. **(I)** Correlation between Notin and classical consensus molecular subtypes (CMSs).

For decades, the functional impact of the Notch pathway-based interplay with lncRNAs for neoplastic diseases has gradually become the focus of researchers’ attention. In this context, we proposed whether a Notch pathway-derived global lncRNA signature could improve the outcomes and treatment efficacy and seek potential drugs for CRC patients. To address the question, we initially validated the Notch index of CRC primary and normal tissues in TCGA-CRC (for training dataset) cohort. Accordingly, the Notch signaling pathway was upregulated in tumor tissues (**Figure 2A**). Further, we utilized single-sample GSEA (ssGSEA) to evaluate the index of Notch pathway features for each sample. K-M analysis showed that the high Notch index predicted the adverse overall survival (OS, log-rank *P* = 0.0001) and relapse-free survival (RFS, log-rank *P* = 0.0003) of patients (**Figures 2B, C**). In addition, correlation analysis between the Notch index and clinical features showed that a high Notch index tended to be associated with a high clinical stage (such as AJCC stage, T, N, and M stage) and microsatellite stability (all *P* < 0.05, **Figures 2D-H**). Notably, the establishment of consensus molecular subtypes (CMSs) provides the most reliable classification system for CRC to date (38). We further explored the underlying link between the Notch index and CMS1–4 and found that CMS4 (mesenchymal subtype) had the highest Notch index (*P* < 0.001, **Figure 2I**). The CMS4 subtype prominently manifested an upregulation of epithelial–mesenchymal transition, angiogenesis, and lowest survival. Collectively, the above support that the Notch pathway has profound predictive value in CRC.

### Nlncer to NLncS: Identifying Notch-derived LncRNA and generating a signature with the LASSO algorithm

**Figure 3:**
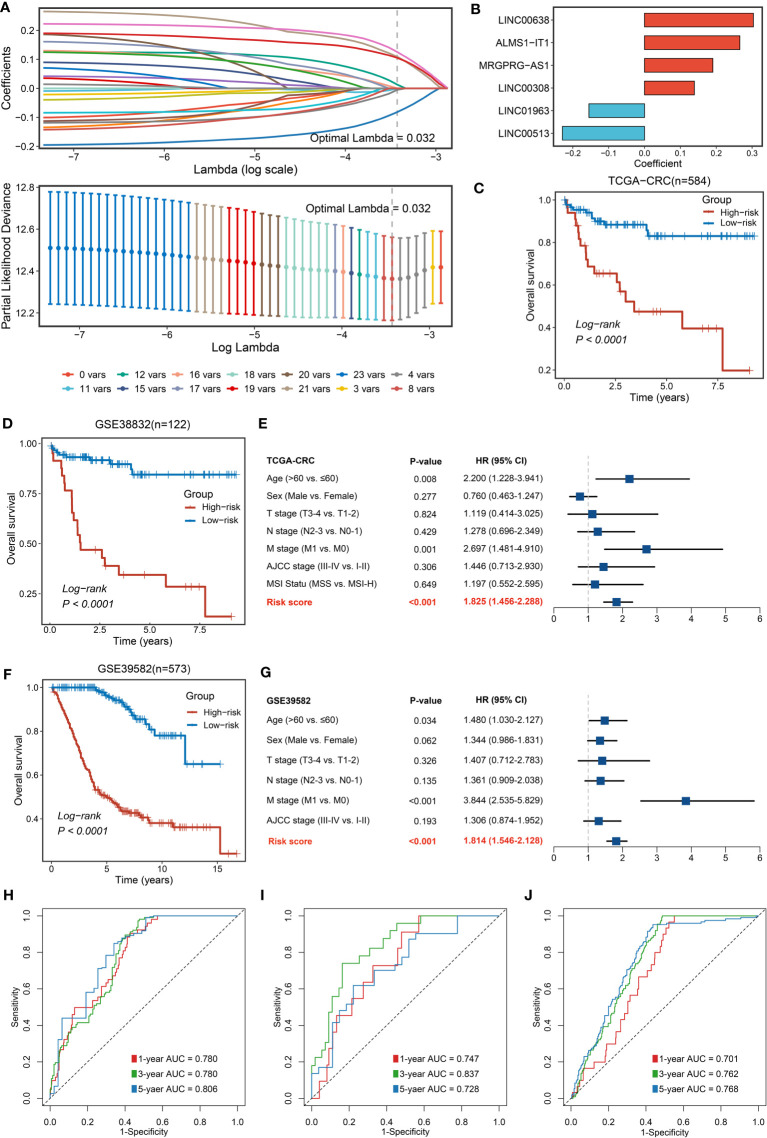
Construction and validation of NLncS. **(A)** Determination of the optimal lambda was obtained when the partial likelihood deviance reached the minimum value. **(B)** Univariate Cox regression was further performed to generate the key lncRNAs with non-zero coefficients and predictive values. LASSO coefficient profiles of the candidate lncRNAs for NLncS construction. **(C, D, F)** Kaplan–Meier curves of OS according to NLncS in TCGA-CRC **(C)**, GSE38832 **(D)**, and GSE39582 **(F)**. **(E, G)** Multivariable Cox regression analysis of NLncS in TCGA-CRC **(E)** and GSE39582 **(G)**. **(H-J)** Time-dependent ROC analysis for predicting OS at 1, 3, and 5 years in TCGA-CRC **(H)**, GSE38832 **(I)**, and GSE39582 **(J)**.

For better consistency, we retained a total of 3,390 lncRNA molecules with relevant data in the three cohorts. Based on our previously developed framework of “TGFmitor” and the recognition of an immune-related lncRNA signature, similarly, we screened and obtained 24 candidate lncRNAs stably associated with the Notch pathway (27, 39, 40). According to the expression profiles of these 24 lncRNAs, we constructed the LASSO model *via* a 10-fold cross-validation. When the optimal lambda of 0.032 was selected, eight key lncRNA molecules with non-zero coefficients were identified (**Figure 3A**). Subsequently, after multivariate Cox regression, six lncRNAs stably associated with OS were used to construct the model, namely, LINC00638, ALMS1-IT1, MRGPRG-AS1, LINC00308, LINC01963, and LINC00513 (**Figure 3B**). Finally, a continuous risk score of the NLncS model was computed through a linear combination of regression coefficient-weighted expression values of the six lncRNAs. Next, we distinguish the high- and low-risk samples according to the median value. In TCGA-CRC, GSE38832, and GSE39582, the high-risk group presented a prominently worse prognosis than the low-risk group (all Log-rank *P <*0.0001, **Figures 3C, D, F**). After incorporating available clinical characteristics including age, sex, stage (T, N, M, and AJCC), and microsatellite instability (MSI) state, multivariate Cox regression showed that NLncS were still statistically significant. This suggests that NLncS can be used as an independent prognostic factor for CRC (all P < 0.001, **Figures 3E, G**, **Supplementary Figure 1A**). We detected the recognition of NLncS and calculated the AUC at 1, 3, and 5 years of TCGA-CRC (0.780, 0.780, and 0.806), GSE38832 (0.701, 0.762, and 0.768), and GSE39582 (0.701, 0.762, and 0.768). We compared the NLncS model with three other lncRNA signatures. It was found that our NLncS model had the highest C-index and was significantly better than the other three lncRNA prediction models (*P < 0.05, ****P < 0.0001, **Supplementary Figure 3**, **Supplementary Table 4**) **(** 41–43). Therefore, NLncS possessed the ability of robust prediction in CRC patients.

### Clinical in-house cohort for NLncS validation *via* qRT-PCR

**Figure 4:**
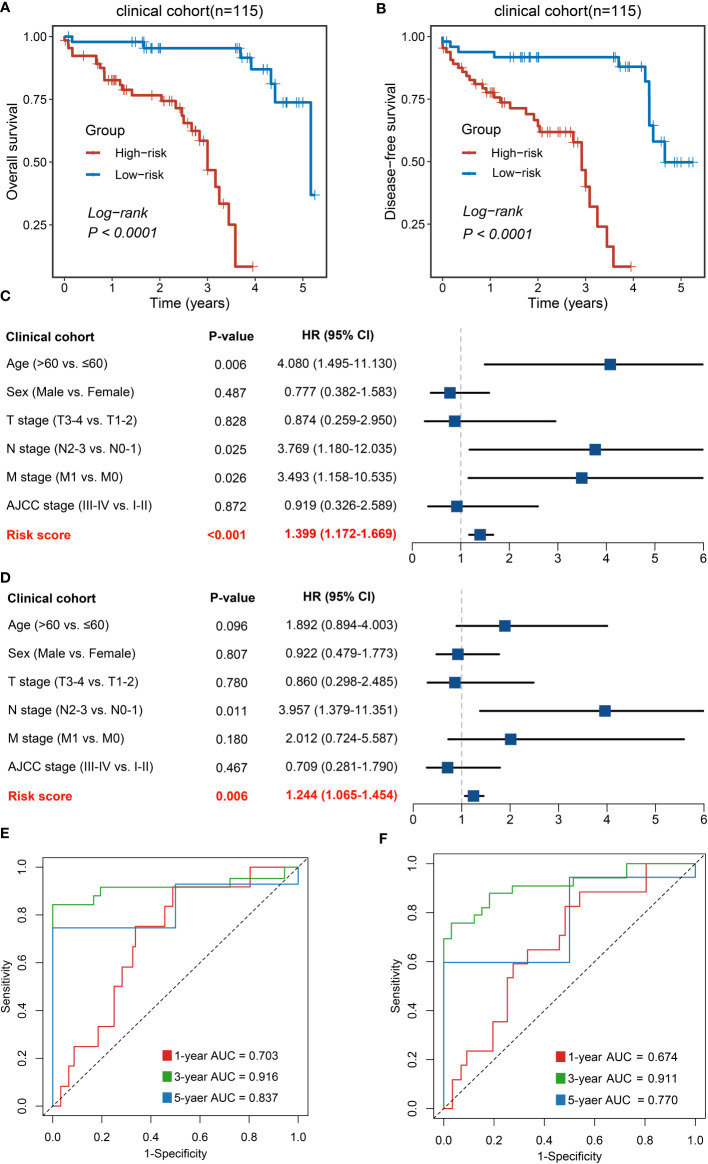
Validation of NLncS *via* qRT-PCR. **(A, B)** Kaplan–Meier curves of OS **(A)** and DFS **(B)** based on NLncS. **(C, D)** Multivariable Cox regression analysis of OS **(C)** and DFS **(D). (E, F)** Time-dependent ROC analysis for predicting OS **(E)** and DFS **(F)** at 1, 3, and 5 years.

To further explore the potential for clinical translational applications of NLncS, we examined the expression of lncRNAs *via* qRT-PCR and calculated risk scores in a clinical in-house cohort. The results are plotted in **Figure 4**. Patients with high-risk scores had lower OS (**Figure 4A**) and disease-free survival (DFS, **Figure 4B**), which validates the prognostic predictive power of the model (*P* < 0.0001 for both). After inclusion of clinical and pathological features, multivariate analysis showed that risk score was an independent indicator in both OS (**Figure 4C**, *P* < 0.001) and DFS (**Figure 4D**, *P* = 0.006). What is more, the AUC calculation of the model showed that the 1-, 3-, and 5-year AUC values were 0.703, 0.916, and 0.837 for OS and 0.674, 0.911, and 0.770 for DFS (**Figures 4E, F** **)**, respectively, showing good predictive power.

### Immune-related mechanisms were upregulated in the high-risk group, showing potential immunologic properties

**Figure 5:**
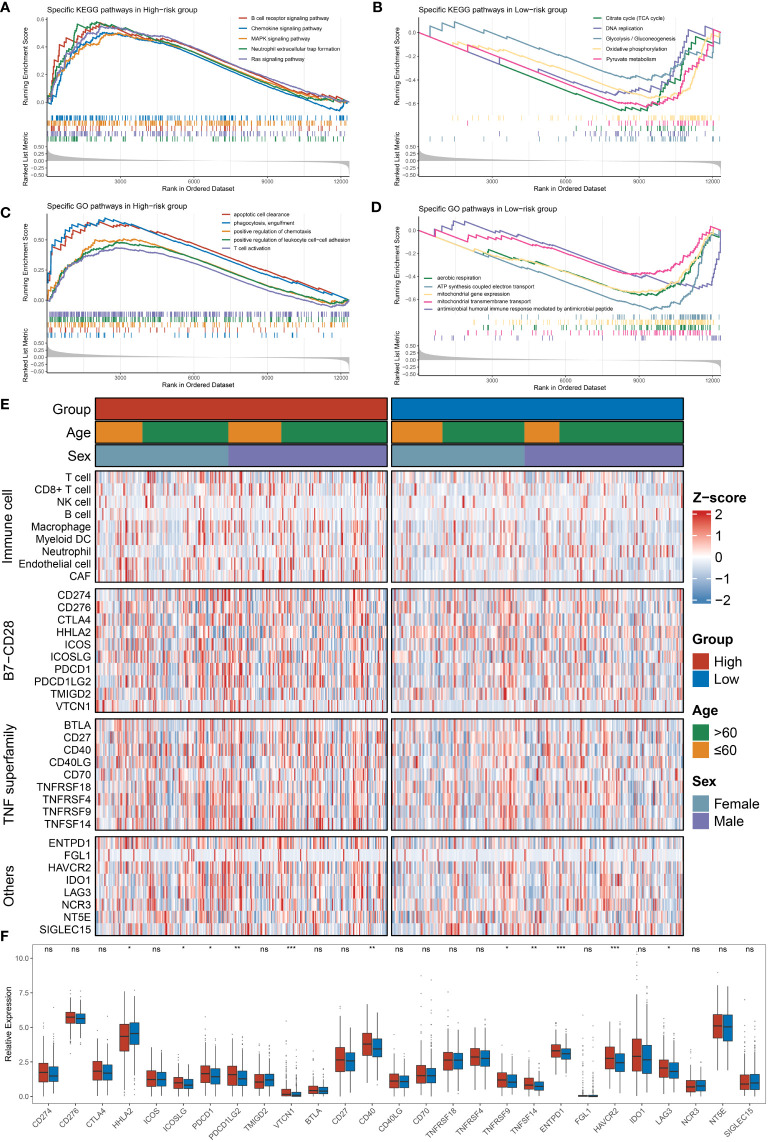
Functional enrichment analysis and immune infiltration analysis were performed in high- and low-risk groups. **(A, B)** The top five KEGG-enriched pathways and immune landscapes in the high- and low-risk groups. **(C, D)** The top five GO-enriched pathways and immune landscapes in the high- and low-risk groups. **(E)** The heatmaps of eight immune cell and 27 immune checkpoint profiles in the high- and low-risk groups. **(F)** The relative expression of 27 immune checkpoints in the high- and low-risk groups. (ns, none significance, P> 0.05; *P< 0.05; **P< 0.01; ***P< 0.001).

Further, we performed GSEA to explore the potential biological mechanisms that may cause differences on two groups. We found a significant enrichment of signaling pathways in the high-risk group such as immunity (humoral and cellular immunity) and regulation of cellular differentiation (**Figures 5A, C** **)**, for instance, B-cell receptor and chemokine signaling, T-cell activation and phagocytosis, and cellular differentiation-related pathways such as the MAPK pathway. However, the downregulation of energy metabolism and redox reactions was predominantly related with the low-risk group, including glycolysis, tricarboxylic acid cycle (TCA cycle), aerobic respiration, and oxidative phosphorylation (**Figures 5B, D** **)**. Hence, it is manifest that immune-related roles may be influencing factors contributing to NLncS predicting differences.

### Tumor immune microenvironment landscape and immune checkpoint profiles of NLncS

Since immune-related pathways differ among subgroups of NLncS, we expanded our analysis of the tumor immune microenvironment (TIME) landscape consisting of nine immune cell types and 27 immune checkpoints to further distinguish immune features. The scenario is depicted in **Figure 5E**. Moreover, CD8+ T cells, macrophages, endothelial cells, and cancer-associated fibroblasts (CAFs) were significantly enriched in the high-risk group (**Supplementary Figure 1B**). Specifically, HHLA2 was overexpressed in the low-risk group, while ICOSLG, PDCD1, PDCD1LG2, VTCN1, CD40, TNFRSF9, TNFSF14, ENTPD1, HAVCR2, and LAG3 were considerably overexpressed in the high-risk group (**Figure 5F**). Thus viewed, both at the level of pathway enrichment, cell infiltration, and molecular expression, the high- and low-risk groups showed more or less imparities.

### Somatic mutation landscape, CNVs, and latent methylation driver in CRC

**Figure 6:**
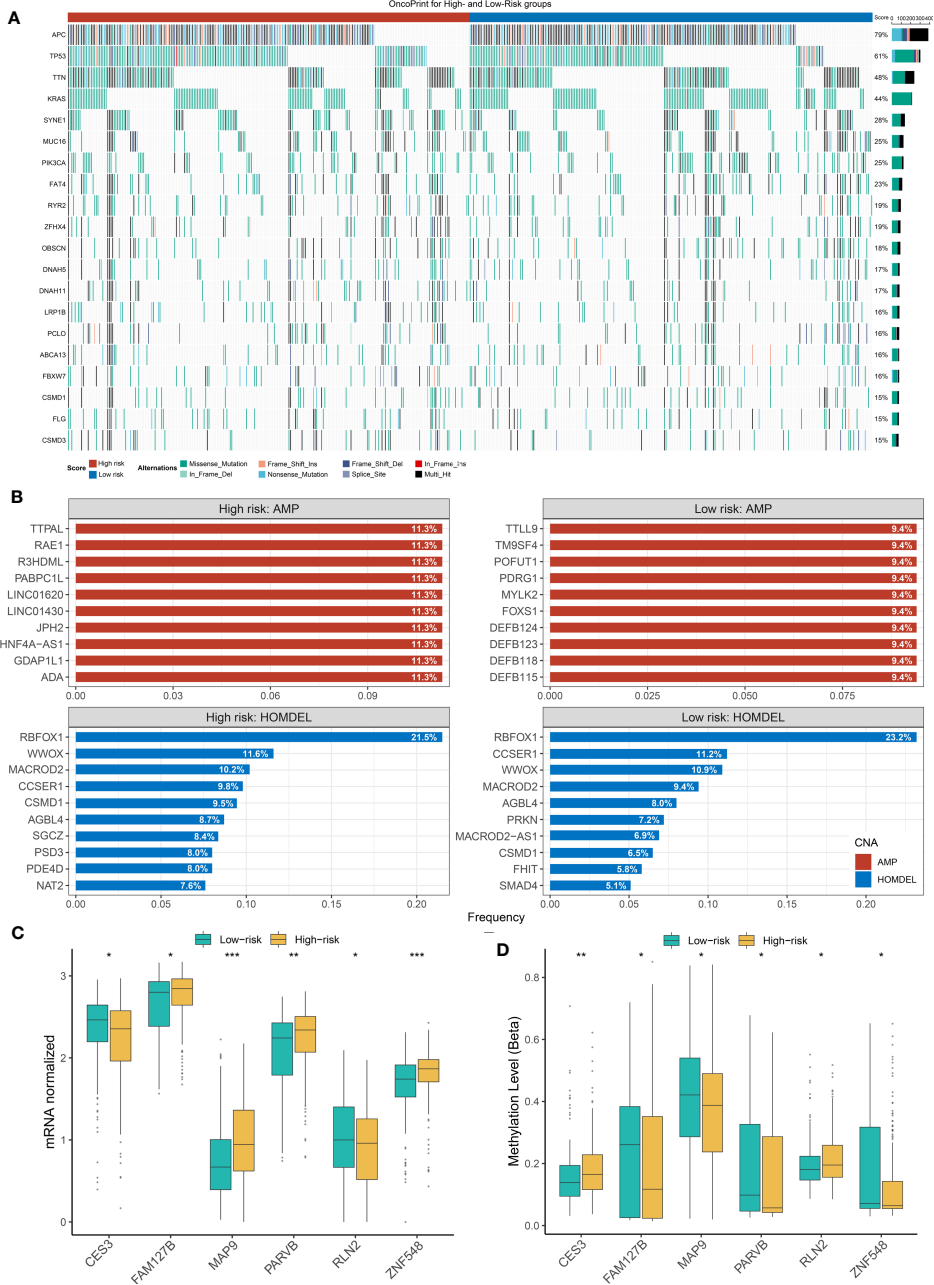
The Landscapes of frequently mutated genes (FMGs) and methylation level in two groups of NLncS. **(A)** The oncoplot depicts the discrepancies in FMGs of CRC among the three cohorts. **(B)** The top 10 genes were amplified and homozygously deleted in the high- and low-risk groups. **(C, D)** The expression and methylation level of the methylation drivers in CRC. (*P< 0.05; **P< 0.01; *** P< 0.001).

Previously, it has been reported that tumors with a high mutation load were more likely to respond to clinical strategies based on immune checkpoint blockers (44–47). Indeed, based on the immunological background of NLncS, we next estimated the effects of somatic mutation on NLncS for seeking clinical benefit. **Figure 6A** depicts the mutational landscape of NLncS. APC and TP53 ranked first and second with 79% and 61% mutation frequencies, respectively, which supported that high mutation rates of APC and TP53 might be responsible for giving rise to CRC (**Figure 6A**). Given that CNV dominatingly consisted of amplification (AMP) and homozygous deletion (HOMDEL), we sequenced the genes by frequency of AMP and HOMDEL (**Figure 6B**). The results revealed that in the high-risk group, TTPAL, RAE1, R3HDML, PABPC1L, LINC01620, and LINC01430 were visibly amplified; dramatically deletions were RBFOX1, WWOX, and MACROD2. Interestingly, there was merely a slight difference in HOMDEL of the low-risk group. Visible amplifications were TTLL9, TM9SF4, POFUT1, PDRG1, MYLK2, and FOXS1.

Considering that the methylation modifications could alter the biological function of RNA molecules at the epigenetic stage, we performed a global methylation overview of CRC (48, 49). With the help of the *MethylMix* R package and Wilcox test, we found six methylation driver genes whose methylation levels were significantly negatively correlated with expression in two groups, including CES3, FAM127B, MAP9, PARVB, RLN2, and ZNF548. Strikingly, the growing NLncS fraction was accompanied by increased methylation levels of CES3 and RLN2 but decreased expression levels (**Supplementary Figure 2**; **Figures 6C, D**). This suggested that CES3 and RLN2 might play an antitumor role as protective factors in the high-risk group, while methylation modification silenced the corresponding mRNA fragments resulting in CES3 and RLN2 reduced expression levels. Collectively, this suggested that the influence of mutational burden, CNV, and methylation level on NLncS scores was likely non-redundant.

### Deducing response to immunotherapy

**Figure 7:**
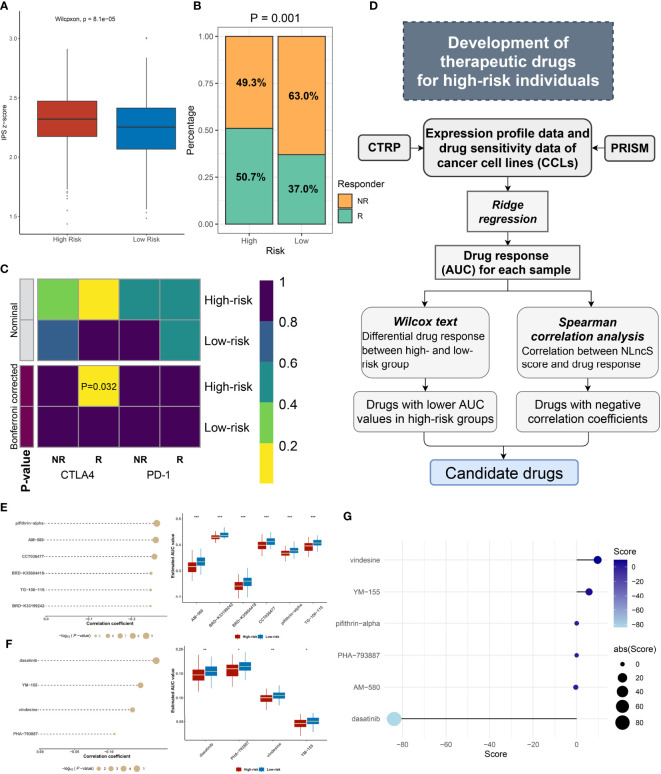
Efficacy evaluation of immunotherapy and potential drug screen for CRC patients. **(A)** The IPS z-score of the two groups. **(B)** The predicted efficacy of the two groups to immunotherapy in TCGA cohorts via TIDE method. **(C)** SubMap analysis of the two groups and 47 pretreated patients with comprehensive immunotherapy annotations in TCGA cohort. For SubMap analysis, a smaller P-value implied a higher similarity of paired expression profiles. **(D)** The framework of exploring insidious drugs for high-risk patients. **(E, F)** The candidate drugs screened by CTRP and PRISM datasets. The correlation analysis of drugs and high-risk groups in CTRP **(E** left**)** and PRISM **(F** left**)**. The differential drug response between high- and low-risk groups in CTRP **(E** right**)** and PRISM **(F** right**)**. **(G)** CMap analysis identified dasatinib as a potential compound able to target the high-risk patient. (*P< 0.05; **P< 0.01; ***P< 0.001).

As we all predicted, immunogenicity predicts stimulation to the immune system (50). As an evaluation tool of tumor immunogenicity, IPS was adopted to indirectly judge the local immune activation status of the sample. Scores were calculated for four different immunophenotypes (antigen presentation, effector cells, suppressor cells, checkpoints). The Z-score was the integration of the four. As expected, we found a significantly high Z-score in high-risk patients, indicating greater immunogenicity (*P <*0.0001, **Figure 7A**). Guided by the TIDE network tool, the response rate to immunotherapy (50.7%) of the high-risk group was markedly higher (*P* = 0.001, **Figure 7B**). Further, according to the SubMap method, we compared the similarity in mRNA expression patterns between two groups of CRC patients and 47 patients who responded (R) or did not respond (NR) to immunotherapy. The result showed that high-risk patients were closer to those who responded to immunotherapy (Bonferroni corrected *P* = 0.032, **Figure 7C**). These results strongly indicate that high-risk individuals could benefit from immunotherapy.

### Exploring insidious therapeutic agents for high-risk CRC individuals

Herein, referring to the workflow of Yang et al., we mined optional drugs for high-risk patients following the framework shown in **Figure 7D** (51). In order to obtain robust therapeutic drugs, we used two databases that store gene expression profile data and drug sensitivity data for CCLs, CTRP, and PRISM. After removing drugs with >20% missing values, we obtained 266 and 1285 compounds from CTRP and PRISM, respectively. We selected ridge regression to predict the drug response for each patient and calculated the AUC for each compound, with a lower AUC suggesting increased sensitivity. Then, we evaluated the appropriate drug in terms of the difference in response and correlation analysis (**Figures 7E, F** **)**. For the difference analysis, we set a strict threshold Log2 FC >0.01 (CTRP) and Log2 FC >0.05 (PRISM), respectively, to identify compounds with lower AUC values, which may act as potential clinical therapies for high-risk individuals. We employed coefficients of r <−0.2 (CTRP) and r <−0.1 (PRISM) to constrain compounds that are more closely related to the NLncS score. In **Figures 7E, F**, a total of six compounds from CTRP and four compounds from PRISM were finally determined (all *P* < 0.05). Further, to more deeply explain the functional links between compounds and RNA molecules as well as disease states, we performed the CMap analysis to explore available drugs (37, 52). Surprisingly, we found that dasatinib, widely used as a first-line antineoplastic agent for leukemia, had a high negative correlation, indicating that it probably to exerts therapeutic effects for high-risk score patients (**Figure 7G**).

## Discussion

As early as 1917, Morgan and colleagues first discovered the Notch gene in mutant flies, and then the Notch signaling pathway was gradually reported (53). Notch signaling is a classical pathway for tumorigenesis and disease progression by accommodating cell proliferation and differentiation, among others (54). Multiple Notch receptors exhibit oncogenic or tumor-suppressive effects in various cells (55). Many clinical trials have also assessed the anticancer efficacy of Notch inhibitors (56). Notably, the interplay of lncRNA molecules with Notch pathway-related molecules regulates a variety of malignant phenotypes of tumors (23–25). Therefore, this study is based on the lncRNA profiles from Notch signaling and aims to establish a model with the ability to predict prognosis and immune response and assist in the screening of sensitive drugs.

The symptoms of CRC are insidious and often lead to the majority of patients presenting at an advanced stage, which contributed to an unfavorable outcome (57). Presently, with the arrival of the era of individualized treatment, the stratification and refined management system for clinical patients urgently need to be improved, and CRC patients are no exception. However, current clinical measures are immature, which is reflected in the inaccuracy of disease prognosis evaluation.

Single biomarkers are also increasingly unable to meet the needs of practical applications, and more and more attention has been paid to synthesizing multiple data types to form appropriate scoring rules (58). We developed an evaluation system consisting of six key lncRNA molecules by LASSO algorithm and Cox regression analysis to perform a risk score that can be continuously quantified in CRC patients. The model showed good prognostic evaluation ability in three public cohorts and one clinical cohort, which can accurately distinguish the high-risk group to facilitate the layer management for clinical patients. This was also initially validated in an internal cohort of 115 clinical individuals.

Consistently, how to evaluate the efficacy of ICP therapy is a problem faced by the majority of oncologists (59–61). On the one hand, “magic drugs” will bring a resurrection effect to some patients, but on the other hand, improper use is likely to cause various side effects (62). Given this, we considered whether the NLncS scoring model could reflect the difference in immune response among distinct patients. We found that there was a significant difference in the pathways enriched. The high-risk group was primarily enriched in cellular or molecular pathways involved in immune processes, including B-cell receptor, chemokine pathways, T-cell activation, and phagocytosis pathways. The low-risk group was primarily downregulated among metabolic aspects (glycolysis, TCA cycle, and oxidative phosphorylation, etc.). Further, immune landscape and immune infiltration analysis suggested visible differences and abundance. Meanwhile, analyses of mutation and methylation levels showed that differences at the genome level were not redundant.

Growing studies have discussed two environments before immune escape occurs in the tumor: 1) ineffective infiltration of a large number of inactive T cells (63); 2) destruction of T cells infiltrating the tumor by immunosuppressive molecules (64). Remarkably, Peng Jiang et al. raised a novel computational architecture, TIDE score, to synthesize these two environments (33). The project involved 189 studies with 33,197 specimens and was thought to be an alternative to a single predictor to analyze the efficacy of immune checkpoint suppression. As predicted, we found that the response rate to immunotherapy in high-risk (50.7%) patients was significantly better than that in low-risk (37.0%) patients (*P* = 0.001). An additional result of SubMap reported that the high-risk group appeared to be more sensitive to CTLA4-blocker-based therapy. These results validate our conjecture that the high-risk group is suitable for immunotherapy.

Dasatinib, as a first-line strategy for metastatic non-small cell lung cancer, can also be widely used as an adjuvant therapy for patients with pancreatic cancer, imatinib-resistant chronic myelogenous leukemia, etc. (65, 66). It is gratifying that we found that dasatinib is an excellent alternative drug for high-risk patients after drug sensitivity prediction and CMap analysis, which not only shows good sensitivity but also reflects powerful targeting ability. However, there is no evidence that dasatinib could be used as a first-line drug in CRC patients yet. Follow-up basic experimental and clinical studies are still needed before dasatinib is adopted to CRC patients. Nonetheless, we argued that dasatinib would possess a bright prospect in improving the prognosis of CRC patients.

Collectively, this study integrally delineated Notch-derived lncRNAs through a “NLncer” workflow and further constructed a systematic scoring system (termed “NLncS”) for accurately and stably evaluating prognosis and immune efficacy in CRC. This work may contribute to interpret patient characteristics and directed therapy. In addition, dasatinib might get a preferential seat in the first line in CRC treatment.

## Data Availability Statement

The original contributions presented in the study are included in the article/**Supplementary Material**. Further inquiries can be directed to the corresponding authors.

## Ethics Statement

The studies involving human participants were reviewed and approved by Ethnics Committee of The First Affiliated Hospital of Zhengzhou University. The patients/participants provided their written informed consent to participate in this study.

## Author Contributions

QD made the conceptualization. ZS, ZL, and YL were involved in the methodology. ZS, LL, CW, and WY provided the resources. QD, LZ, and YL analyzed the data. QD and YL prepared the original draft. QD and WW reviewed and edited the manuscript. ZS, LL, and CW supervised the study. All authors contributed to the article and approved the submitted version.

## Funding

This study was supported by The National Natural Science Foundation of China (81972663, 82173055, U2004112), The Excellent Youth Science Project of Henan Natural Science Foundation (212300410074), The Key Scientific Research Project of Henan Higher Education Institutions (20A310024), The Youth Talent Innovation Team Support Program of Zhengzhou University (32320290), The Provincial and Ministry co-constructed key projects of Henan Medical Science and Technology (SBGJ202102134), Key Scientific and Technological Research Projects of Henan Provincial Department of Science and Technology (212102310117), Henan Provincial Health Commission and Ministry of Health Co-construction Project, and Henan Provincial Health and Health Commission Joint Construction Project (LHGJ20200158).

## Conflict of Interest

The authors declare that the research was conducted in the absence of any commercial or financial relationships that could be construed as a potential conflict of interest.

## Publisher’s Note

All claims expressed in this article are solely those of the authors and do not necessarily represent those of their affiliated organizations, or those of the publisher, the editors and the reviewers. Any product that may be evaluated in this article, or claim that may be made by its manufacturer, is not guaranteed or endorsed by the publisher.

## Supplementary Material

Supplementary Figure 1 The multivariable Cox regression analysis of NLncS in GSE38832 **(A).** And the differences in the abundance of cell infiltrates between high- and low-risk groups **(B)**.

Supplementary Figure 2 Correlation analysis between expression of identified methylation drivers and methylation levels.

Supplementary Figure 3 Comparison of predictive efficacy of NLncS model with three published studies.
