# Supplementary figures and images for: LncRNA profiles from Notch signaling: Implications for clinical management and tumor microenvironment of colorectal cancer
Source: Front Immunol. 2022 Jul 25;13:953405. doi: 10.3389/fimmu.2022.953405 (PMC9359081; doi:10.3389/fimmu.2022.953405)

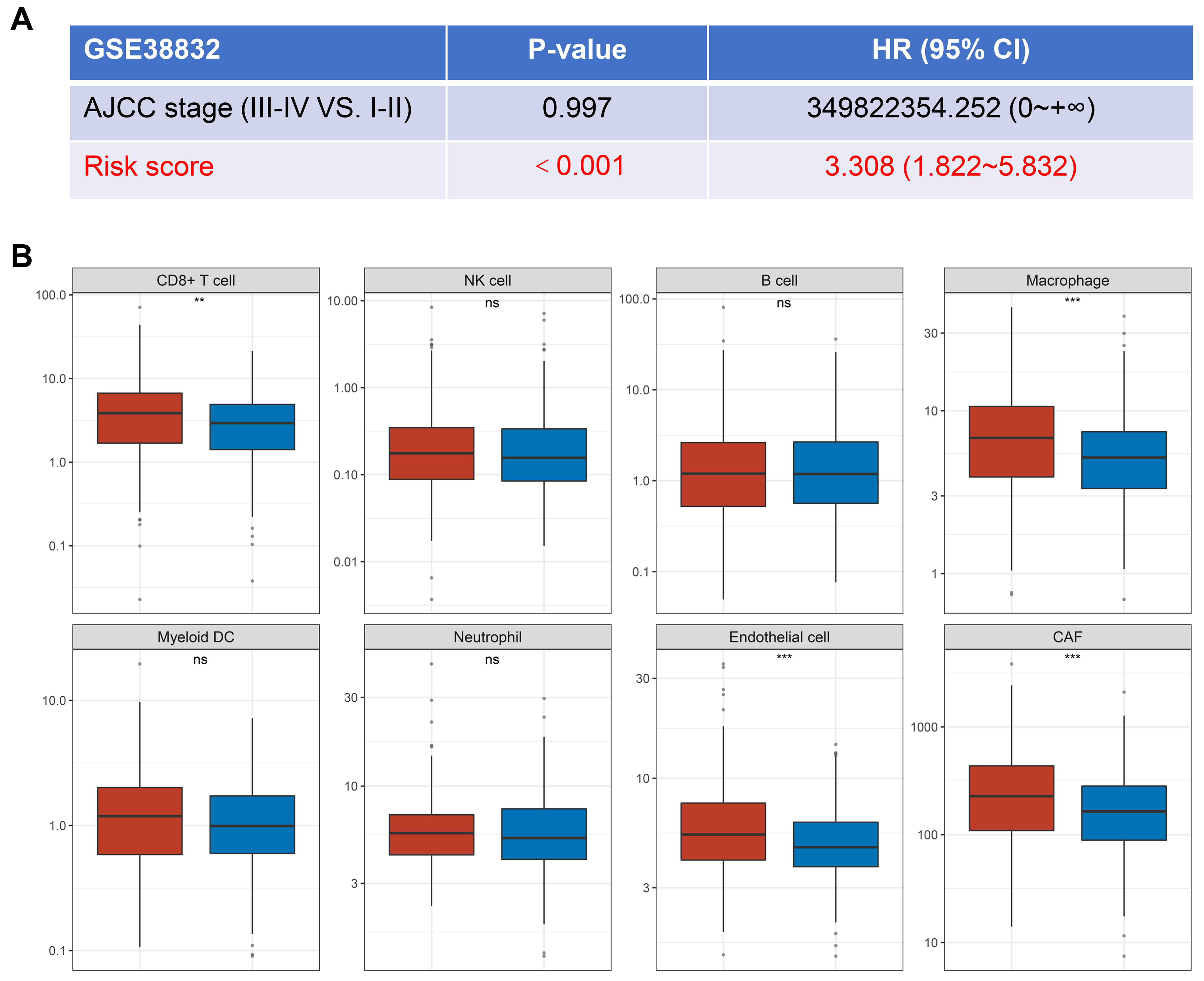

Supplement: Supplementary Figure 1 — The multivariable Cox regression analysis of NLncS in GSE38832 (A). And the differences in the abundance of cell infiltrates between high- and low-risk groups (B). [file Image_1.tif]

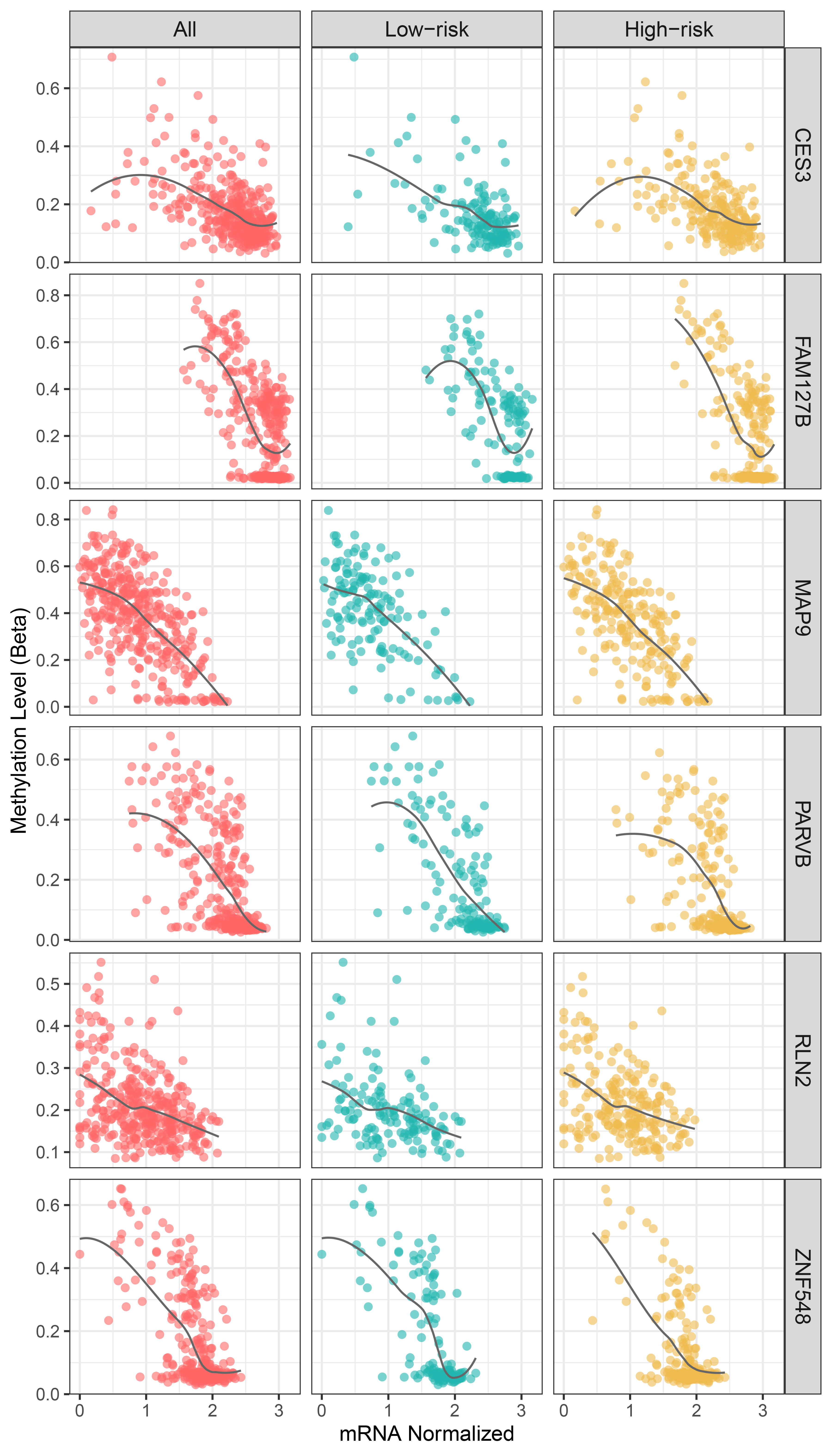

Supplement: Supplementary Figure 2 — Correlation analysis between expression of identified methylation drivers and methylation levels. [file Image_2.tif]

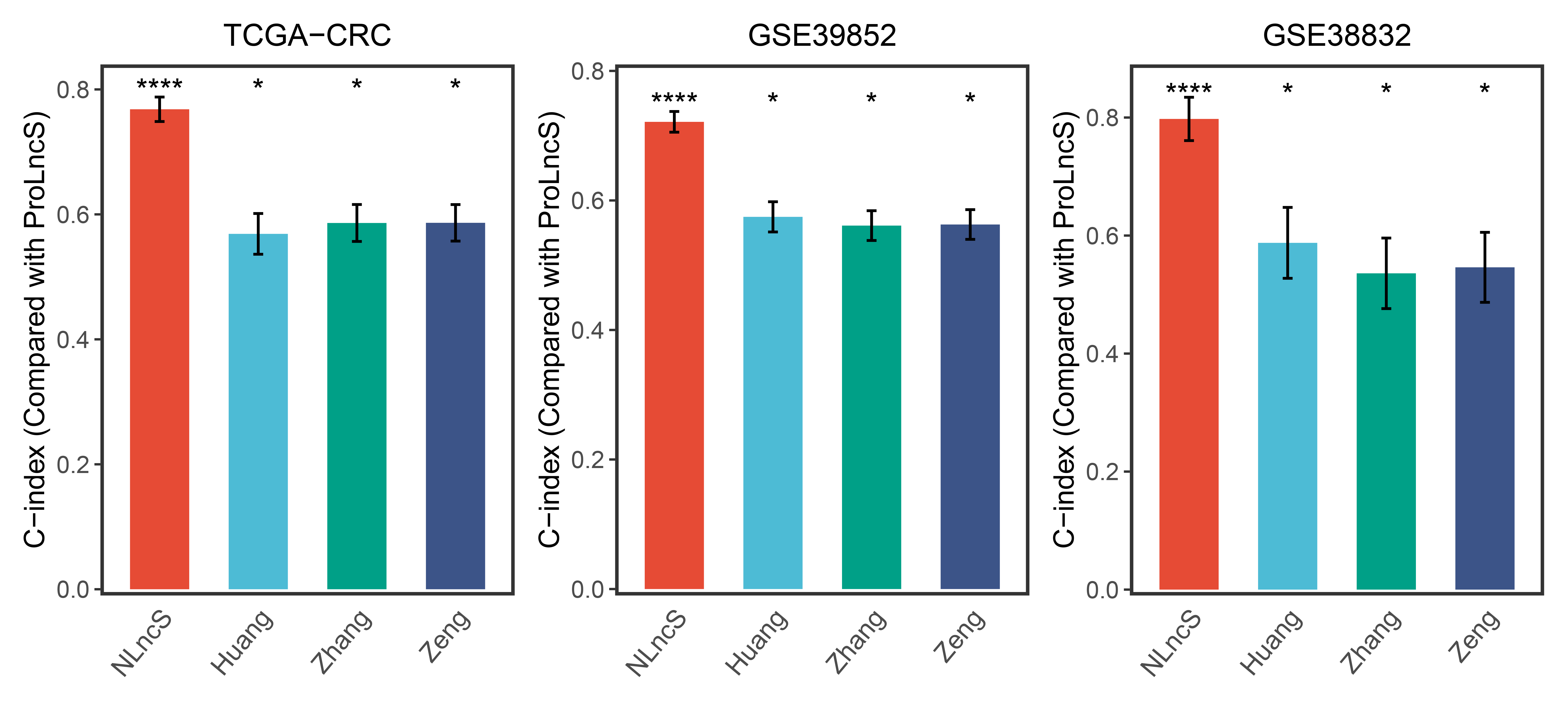

Supplement: Supplementary Figure 3 — Comparison of predictive efficacy of NLncS model with three published studies. [file Image_3.tif]
